# Supplementary material for: The Impact of DNMT3A Status on NPM1 MRD Predictive Value and Survival in Elderly AML Patients Treated Intensively
Source: Cancers (Basel). 2021 Apr 29;13(9):2156. doi: 10.3390/cancers13092156 (PMC8124973; doi:10.3390/cancers13092156)
Supplement: Supplementary file 1 [file cancers-13-02156-s001.zip › cancers-1154534-supplementary.pdf]

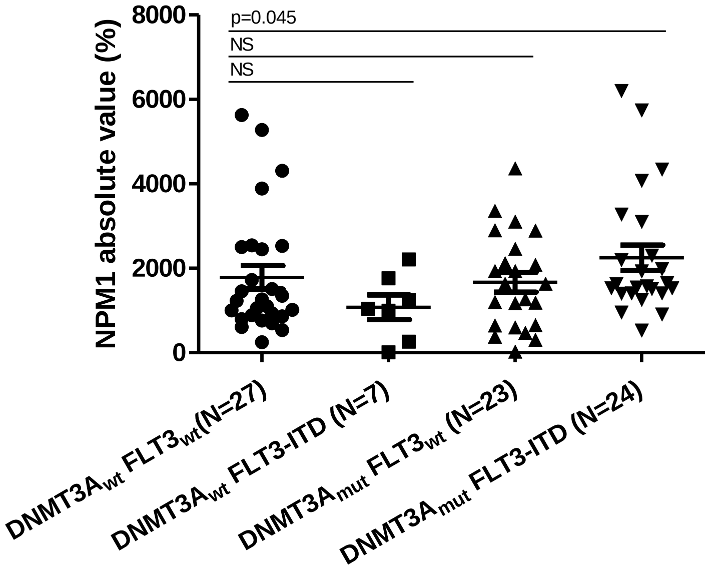

Figure S1: *NPM1* RT-qPCR absolute value according to *FLT3*-ITD and *DNMT3A* mutational status.

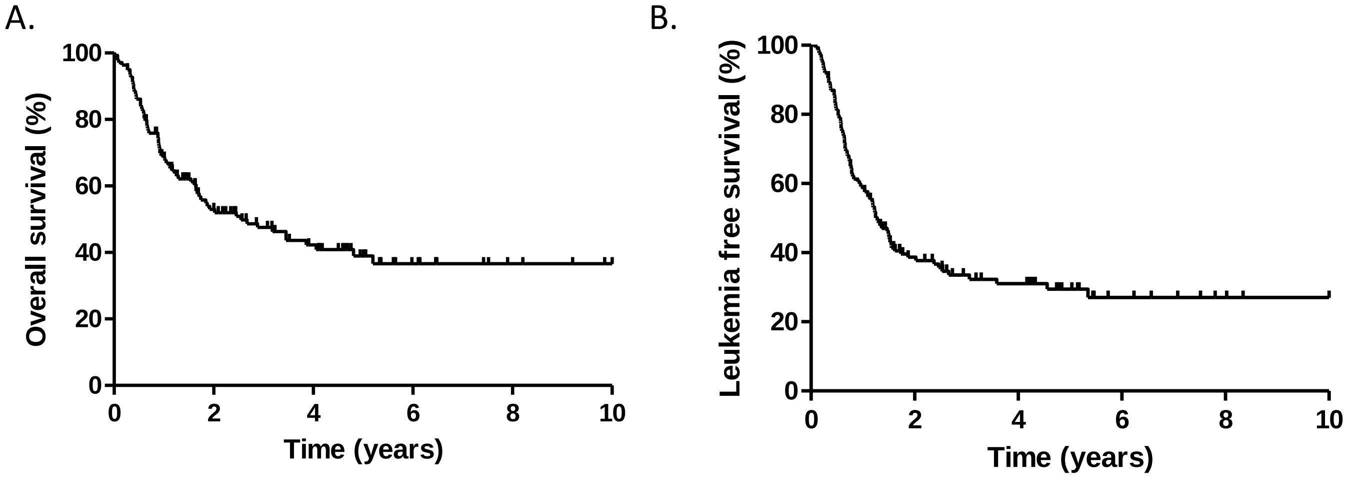

Figure S2: A. Overall survival and B. Leukemia free survival of the entire cohort (N=138)

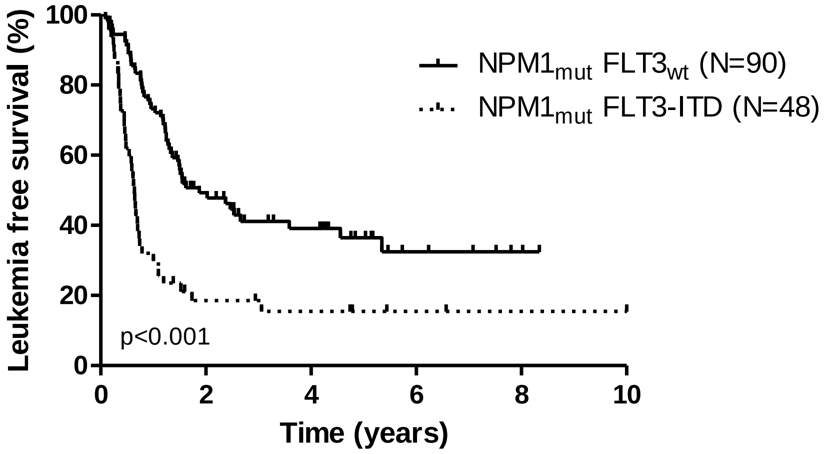

Figure S3: Leukemia free survival according to *NPM1* and *FLT3*-ITD status

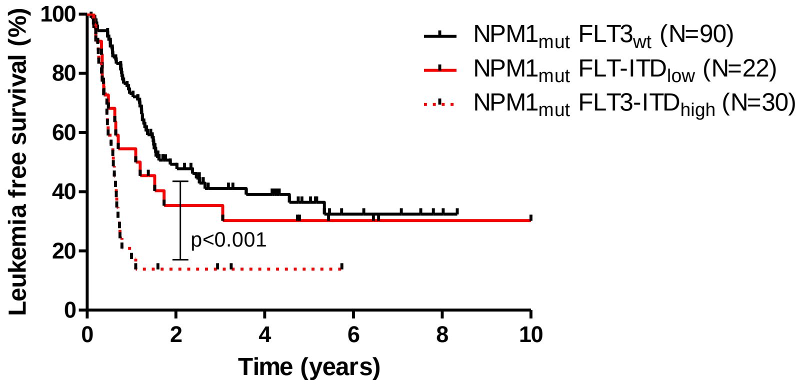

Figure S4: Leukemia free survival according to *FLT3-ITD* allelic ratio

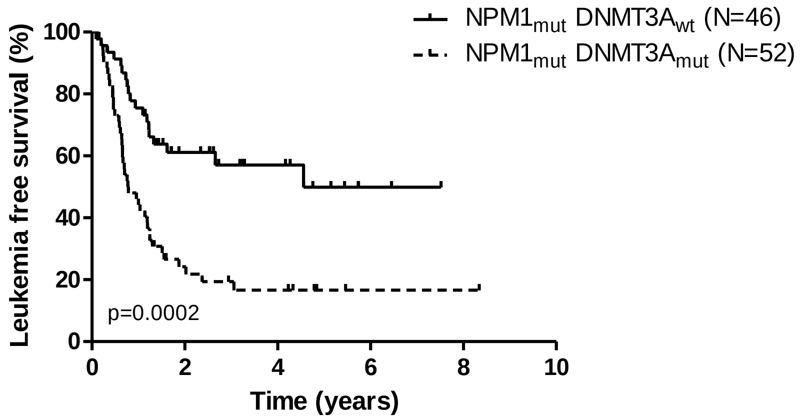

Figure S5: Leukemia free survival according to *DNMT3A* status

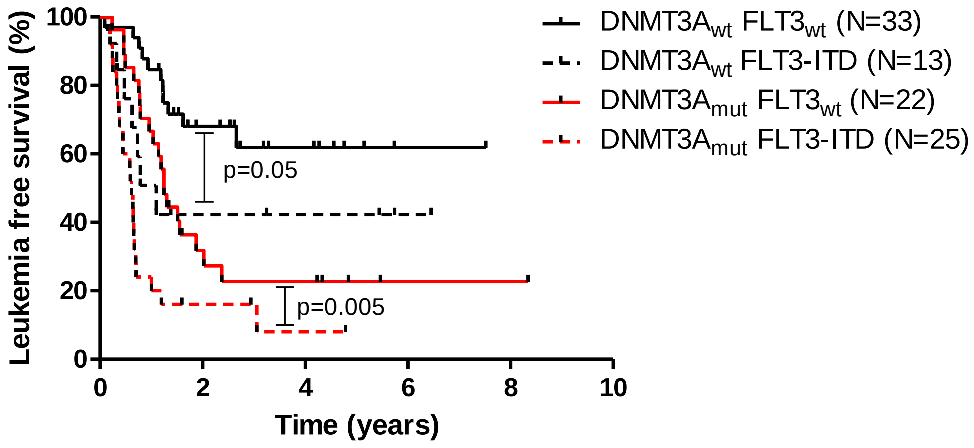

Figure S6: Leukemia free survival according to *DNMT3A and FLT3*-ITD status

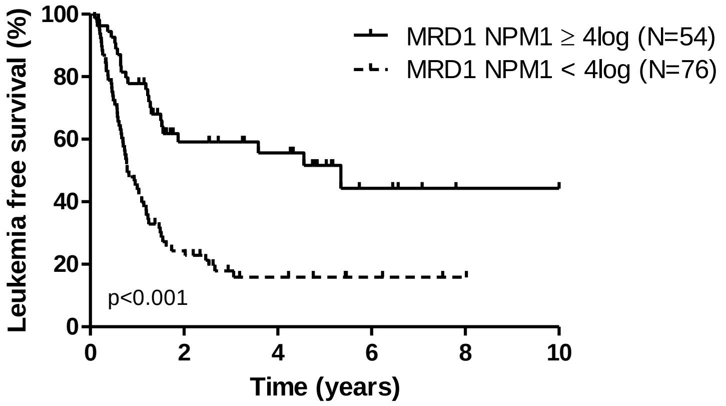

Figure S7: Leukemia free survival according *NPM1* BM-MRD1

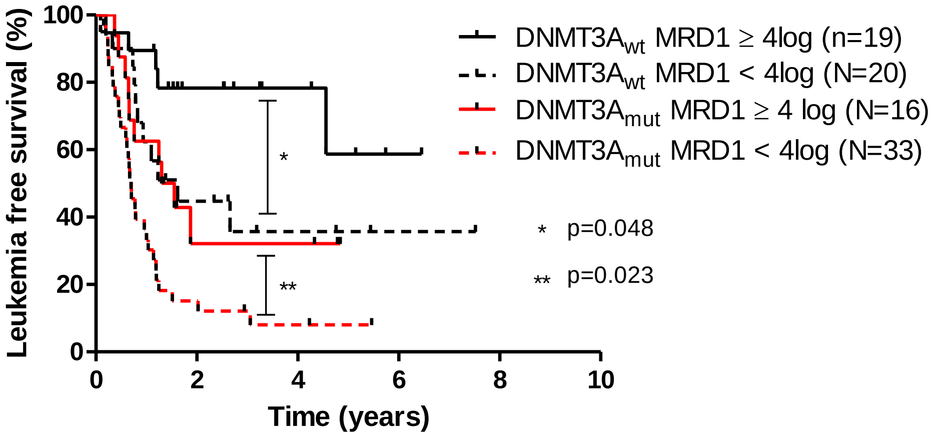

Figure S8: Leukemia free survival according *NPM1* BM-MRD1 and *DNMT3A* status

Variables	Overall survival HR (IC 95%)	P-value	Leukemia free survival HR (IC 95%)	P-value
Age (< 70 vs ≥ 70 yo)	0.95 (0.60-1.51)	0.84	0.83 (0.50-1.34)	0.43
Karyotype (normal vs abnormal)	0.56 (0.27-1.18)	0.13	0.53 (0.27-1.04)	0.061
ELN 2017 (favorable vs other)	0.29 (0.16-0.49)	<0.001	0.29 (0.17-0.48)	<0.001
FLT3-ITD (mut vs wt)	2.52 (1.51-4.22)	<0.001	3.11 (1.903-5.09)	<0.001
DNMT3A (mut vs wt)	2.52 (1.41-4.51)	0.002	2.79 (1.67-4.64")	<0.001
MRD1 ≥ 4 log reduction (yes vs no)	0.35 (0.22-0.57)	<0.001	0.37 (0.24-0.57)	<0.001
HSCT in CR1 (yes vs no)	1.41 (0.71-2.87)	0.31	0.84 (0.48-1.50)	0.57

Table S1: univariate analysis

NPM1 SS risk group	Median OS, months	HR (IC 95%)	P-value	Median LFS, months	HR (IC 95%)	P-value
Favorable	NR	1	-	NR	1	-
Intermediate	30.6	0.48 (0.23-0.85)	0.019	17.2	0.45 (0.26-0.77)	0.004
Unfavorable	13.2	0.23 (0,14-0,48)	<0.001	7.7	0.14 (0.07-0.29)	<0.001

Supplemental Table 2: Overall survival and leukemia free survival according to NPM1 SS subgroups
